# Education interacts with genetic variants near *GJD2*, *RBFOX1*, *LAMA2*, *KCNQ5* and *LRRC4C* to confer susceptibility to myopia

**DOI:** 10.1371/journal.pgen.1010478

**Published:** 2022-11-17

**Authors:** Rosie Clark, Alfred Pozarickij, Pirro G. Hysi, Kyoko Ohno-Matsui, Cathy Williams, Jeremy A. Guggenheim, UK Biobank Eye and Vision Consortium

**Affiliations:** 1 School of Optometry & Vision Sciences, Cardiff University, Cardiff, United Kingdom; 2 Section of Ophthalmology, School of Life Course Sciences, King’s College London, London, United Kingdom; 3 Department of Twin Research and Genetic Epidemiology, School of Life Course Sciences, King’s College London, London, United Kingdom; 4 Department of Ophthalmology and Visual Science, Tokyo Medical and Dental University, Tokyo, Japan; 5 Centre for Academic Child Health, Population Health Sciences, Bristol Medical School, University of Bristol, Bristol, United Kingdom; Case Western Reserve University, UNITED STATES

## Abstract

Myopia most often develops during school age, with the highest incidence in countries with intensive education systems. Interactions between genetic variants and educational exposure are hypothesized to confer susceptibility to myopia, but few such interactions have been identified. Here, we aimed to identify genetic variants that interact with education level to confer susceptibility to myopia. Two groups of unrelated participants of European ancestry from UK Biobank were studied. A ‘Stage-I’ sample of 88,334 participants whose refractive error (*avMSE*) was measured by autorefraction and a ‘Stage-II’ sample of 252,838 participants who self-reported their age-of-onset of spectacle wear (*AOSW*) but who did not undergo autorefraction. Genetic variants were prioritized via a 2-step screening process in the Stage-I sample: Step 1 was a genome-wide association study for *avMSE*; Step 2 was a variance heterogeneity analysis for *avMSE*. Genotype-by-education interaction tests were performed in the Stage-II sample, with University education coded as a binary exposure. On average, participants were 58 years-old and left full-time education when they were 18 years-old; 35% reported University level education. The 2-step screening strategy in the Stage-I sample prioritized 25 genetic variants (GWAS *P* < 1e-04; variance heterogeneity *P* < 5e-05). In the Stage-II sample, 19 of the 25 (76%) genetic variants demonstrated evidence of variance heterogeneity, suggesting the majority were true positives. Five genetic variants located near *GJD2*, *RBFOX1*, *LAMA2*, *KCNQ5* and *LRRC4C* had evidence of a genotype-by-education interaction in the Stage-II sample (*P* < 0.002) and consistent evidence of a genotype-by-education interaction in the Stage-I sample. For all 5 variants, University-level education was associated with an increased effect of the risk allele. In this cohort, additional years of education were associated with an enhanced effect of genetic variants that have roles including axon guidance and the development of neuronal synapses and neural circuits.

## Author summary

Myopia (short-sightedness) is a refractive error that typically develops during childhood. Uncorrected myopia causes blurred distance vision. Myopia is also associated with a range of eye disorders, making it a leading cause of irreversible visual impairment in older age. About 80% of children in East Asia develop myopia during school-age; in the West the corresponding figure is about 30%. Genetics, insufficient time spent outdoors, and years spent in education are risk factors for myopia. Genetics studies have identified more than 450 genetic variants associated with an increased risk of myopia. However, very few genetic variants have been found that confer an increased risk of myopia specifically in individuals exposed to higher levels of lifestyle risk factors. Here, we leverage statistical features expected for variants with gene-environment interaction effects and harness the large sample size of UK Biobank, to identify 5 genetic variants that confer a progressively increased risk of myopia in individuals who spent increasing years in education. Two of the variants replicate findings reported in East Asian cohorts, while the other 3 variants are novel. This work provides insight into the biological pathways through which genes and lifestyle interact to cause myopia.

## Introduction

Myopia (short-sightedness) is a refractive error caused by a mismatch between the focal and axial lengths of the eye. Myopia currently affects 22% of the world population and its prevalence is increasing, especially in recent birth cohorts [1,2]. Individuals with myopia are at greater risk of ocular pathologies such as glaucoma, myopic maculopathy and retinal detachment [2,3]. Refractive errors have a significant economic, societal and healthcare impact due to the requirement of sight tests, corrective aids or surgery, and the associated increased risk of blindness and sight impairment [4].

Refractive errors are highly heritable. To date, genome-wide association studies (GWAS) have identified more than 450 genetic variants associated with refractive error [5,6]. Pathway analysis of these gene variants has highlighted a diverse range of functions, signaling pathways and cellular processes involving many different retinal cell types, as well as the structure and function of the extracellular matrix. Environmental factors such as years spent in education, excessive near work, and less time outdoors are also associated with myopia [7–12]. Typically, genetic and lifestyle risk factors for refractive error have been studied separately, with few studies directly assessing the contribution from gene-environment (GxE) interaction effects [13–15].

**Fig 1:**
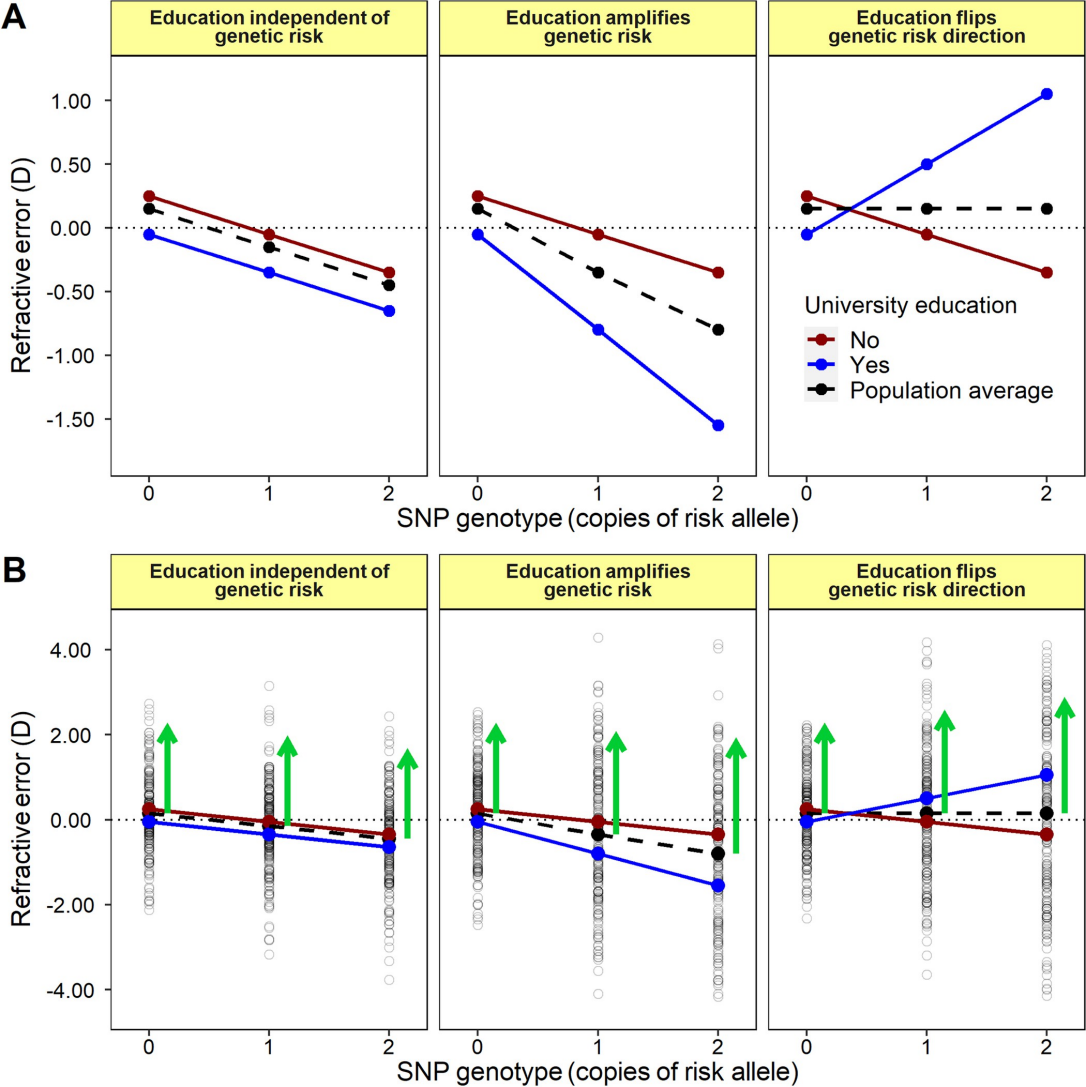
Two-step screening strategy for selecting SNPs likely to be involved in a GxE interaction. The graphs provide schematic illustrations of SNPs without (left) and with (middle and right) GxE interactions, using education as an exemplar environmental exposure. **A**: Standard GWAS analysis will detect effects averaged across environments for the population (black dashed line). Thus, a standard GWAS analysis will detect SNPs that do not have GxE interaction effects (left panel) but it will also detect SNPs that do have GxE interaction effects (middle panel) unless the SNP effects cancel out in different environments (right panel). **B**: SNPs involved in GxE interactions are expected to exhibit variance heterogeneity for each genotype, which is depicted as differences in height of the green arrows (middle and right panels) whereas variance heterogeneity is not expected for a SNP with no GxE interaction (left panel).

A standard GWAS tests for an association between the genotype of single nucleotide polymorphisms (SNPs) and the *mean* level of a phenotype. By contrast, a ‘variance heterogeneity’ analysis tests for SNPs that exhibit a difference in phenotype *variance* across genotypes (Fig 1). Most SNPs with GxE interaction effects are expected to be variance heterogeneity quantitative trait loci (‘vQTLs’) [16]. Notably, information about each participant’s level of exposure to the relevant environmental risk factor is not required to perform a variance heterogeneity analysis; only genotype and phenotype information is required. A variance heterogeneity analysis can be used as a screening step to prioritize SNPs for further assessment for GxE interaction effects, which reduces the multiple testing burden and loss of statistical power associated with testing vast numbers of variants [16–19]. Here, we adopted a 2-step genome-wide screening strategy, comprising of a standard GWAS analysis followed by a variance heterogeneity analysis, to enrich for SNPs with an above-average likelihood of involvement in a GxE interaction associated with refractive error. We then assessed the prioritized SNPs for genotype-by-education interaction effects.

## Results

Analyses were performed in 2 independent samples of UK Biobank participants (Table 1): a ‘Stage-I’ sample (N = 88,334) with known refractive error (*avMSE*) and a ‘Stage-II’ sample (N = 252,838) with known age-of-onset of spectacle wear (*AOSW*). On average, participants were 58 years-old (range 40–73 years) and left full-time education at age 18 years (range 13–26 years). Approximately one third of participants had undergone University-level education (coded as a binary variable, *UniEdu*). The sample size was approximately 3-fold larger for the Stage-II sample than the Stage-I sample due to autorefractor measurements only being performed in the later stage of UK Biobank recruitment.

**Table 1:** Demographic characteristics of the Stage-I and Stage-II samples. Values are means (standard deviations in paratheses).

Trait	Stage-I sample (N = 88,334)	Stage-II sample (N = 252,838)
Female (proportion)	0.53	0.55
Age (years)	57.7 (7.9)	58.2 (7.5)
Age leaving education (years)	18.3 (2.5)	18.0 (2.5)
*UniEdu* (proportion)	0.37	0.32
*AOSW* (years)	32.4 (16.9)	31.6 (17.0)
Myopic (proportion)	0.34	0.38
*avMSE* (D)	-0.25 (2.67)	-

### Overview of the two-step strategy for selecting variants involved in GxE interactions

A 2-step screening strategy in the Stage-I sample, with *avMSE* as the phenotype-of-interest, was used to select a set of genetic variants with above-average likelihood of involvement in a GxE interaction (Fig 1). Step 1 was a standard GWAS for refractive error, testing for a SNP-phenotype ‘marginal effect’. SNPs with GxE interaction effects are expected to show a marginal effect association unless the direction of effect of the SNP reverses at different levels of the environmental exposure (Fig 1A). After ‘clumping’ SNPs in high linkage disequilibrium (LD) to identify independently associated variants, the GWAS identified 956 SNPs with suggestive evidence of association with *avMSE* at the liberal threshold of *P* < 1 x 10^−4^ (S1 Table and Fig A in S1 Text). Step 2 was a test for variance heterogeneity, which is also an expected feature of many of the SNPs involved in a GxE interaction (Fig 1B). Of the 956 independently associated SNPs identified from Step 1, there were 25 variants (3%) with evidence of variance heterogeneity for *avMSE* (variance heterogeneity *P* < 5 x 10^−5^; Bonferroni correction for 956 tests). Simulations confirmed that the variance heterogeneity test (Levene’s median test) maintained the correct type-I error rate under the test conditions, despite the non-normal distributions of *avMSE* (Box A in S1 Text). Details of the 25 variants identified by the 2-step screening strategy are presented in Table 2.

**Table 2:** Genetic variants significantly associated with variance in refractive error (variance heterogeneity). Levene’s median test p-values are given for the tests carried out in the Stage-I sample and the Stage-II sample. Variants are ranked by p-value in the Stage-I sample. Novel genes are indicated; a citation is given for genes previously implicated in myopia development. The effect allele is the allele associated with a more myopic refractive error in a linear regression test for the marginal effect of the variant.

Variant	Nearest Gene	CHR	POS	EA	NEA	FreqEA	*P*-value (Stage-I sample)	*P*-value (Stage-II sample)	Novel
rs634990	GJD2	15	35006073	C	T	0.492	1.18E-17	1.37E-09	[34]
rs11602008	LRRC4C	11	40149305	T	A	0.17	9.31E-16	1.57E-14	[70]
rs12193446	LAMA2	6	129820038	A	G	0.904	1.32E-15	3.08E-28	[70]
rs1550094	PRSS56	2	233385396	G	A	0.304	9.03E-11	3.83E-06	[71]
rs9911460	FAAP100	17	79538841	T	A	0.477	5.68E-10	1.29E-39	Yes
rs7744813	KCNQ5	6	73643289	A	C	0.588	1.59E-09	7.34E-10	[71]
rs7188859	RBFOX1	16	7460426	C	T	0.365	6.91E-09	9.59E-17	[39]
rs10917958	NMNAT1P2	1	164214985	T	C	0.234	2.06E-08	1.40E-05	Yes
rs368893443	ACTN2	13	100690003	AAGAG	A	0.454	3.07E-07	3.85E-08	Yes
rs6979354	SP4	7	21417963	T	C	0.555	1.71E-06	1.40E-02	Yes
rs12450368	CDRT15	17	14138507	C	T	0.409	4.03E-06	3.42E-04	[5]
rs13181905	EBF1	5	158492279	A	C	0.436	6.28E-06	3.51E-02	[72]
rs11033093	SLC1A2	11	35381495	G	C	0.103	7.18E-06	9.98E-01	Yes
rs2980823	TCIM	8	40148843	G	T	0.582	9.54E-06	4.83E-01	Yes
rs36005291	TOX	8	60179048	CA	C	0.655	1.24E-05	2.20E-06	[71]
rs418092	GPX6	6	28533946	T	C	0.333	1.30E-05	3.60E-02	[5]
rs2607006	SGF29	16	28576017	C	T	0.414	1.48E-05	8.89E-01	Yes
rs9518096	GGACT	13	101223109	C	T	0.481	1.74E-05	3.00E-06	[5]
rs77483535	THAP6	4	76312573	T	C	0.302	2.48E-05	3.67E-01	Yes
rs435555	SLC52A1	17	4947305	T	C	0.556	2.83E-05	1.03E-03	Yes
rs34720604	KLHL1	13	70190188	T	TA	0.729	3.40E-05	5.62E-01	Yes
rs869422	ZMAT4	8	40723970	A	G	0.794	3.67E-05	2.20E-12	[71]
rs1868289	GRIN2A	16	10215813	T	G	0.678	3.84E-05	2.79E-02	[73]
rs7678123	CFAP299	4	81372405	C	G	0.284	4.25E-05	5.89E-06	Yes
rs10637890	CDKN3	14	54779523	T	TCT	0.375	4.57E-05	6.71E-01	[5]

Abbreviations: CHR = Chromosome; POS = Genomic position for GRCh37; EA = Effect allele; NEA = Non-effect allele; FreqEA = Allele frequency of effect allele.

### Confirmation of variance heterogeneity in the Stage-II sample

The 25 variants identified using the 2-step screening strategy in the Stage-I sample were examined for evidence of variance heterogeneity in the Stage-II sample for the outcome *AOSW*. Seventy six percent of the variants (19 out or 25) displayed evidence of variance heterogeneity in the Stage-II sample (Levene’s test, *P* < 0.05; Table 2), which was a higher proportion than expected by chance (binomial test, *P* = 2.52 x 10^−20^) and suggested that the majority of the 25 vQTL were true positive findings. Simulations suggested Levene’s test maintained the correct type-1 error rate for the *AOSW* phenotype, despite its highly non-normal distribution (Box A in S1 Text).

### GxE interaction between genetic variants and educational attainment

**Fig 2:**
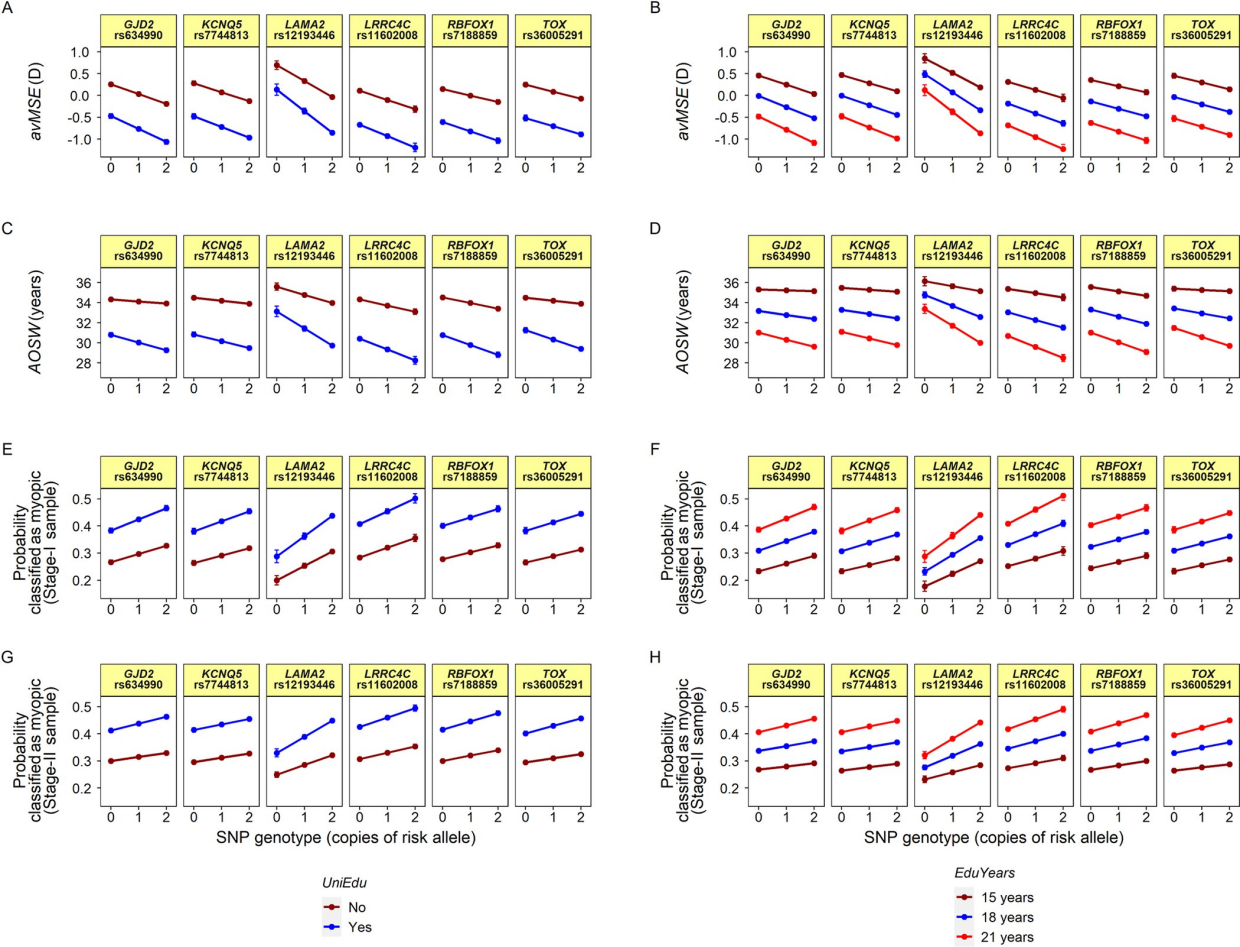
Genetic variants with evidence of genotype-by-education interactions. Results are presented for a range of outcome traits, for tests of the interaction between SNP genotype and education level. Education level is coded as either *UniEdu* (panels A, C, E & G) or *EduYears* (panels B, D, F & H). Panels A & B: Linear regression analysis for the outcome *avMSE* in the Stage-I sample. Panels C & D: Linear regression analysis for the outcome age-of-onset of spectacle wear (*AOSW*) in the Stage-II sample. Panels E & F: Logistic regression analysis for the outcome *Myopic* (calculated based on *avMSE*) in the Stage-I sample. Panels G & H: Logistic regression analysis for the outcome *Myopic* (calculated based on *AOSW*) in the Stage-II sample. The nearest gene to the SNP is indicated above the SNP rsID. Error bars are 95% confidence intervals. The SNP risk allele was defined as the myopia-predisposing allele in a marginal effects analysis.

**Fig 3:**
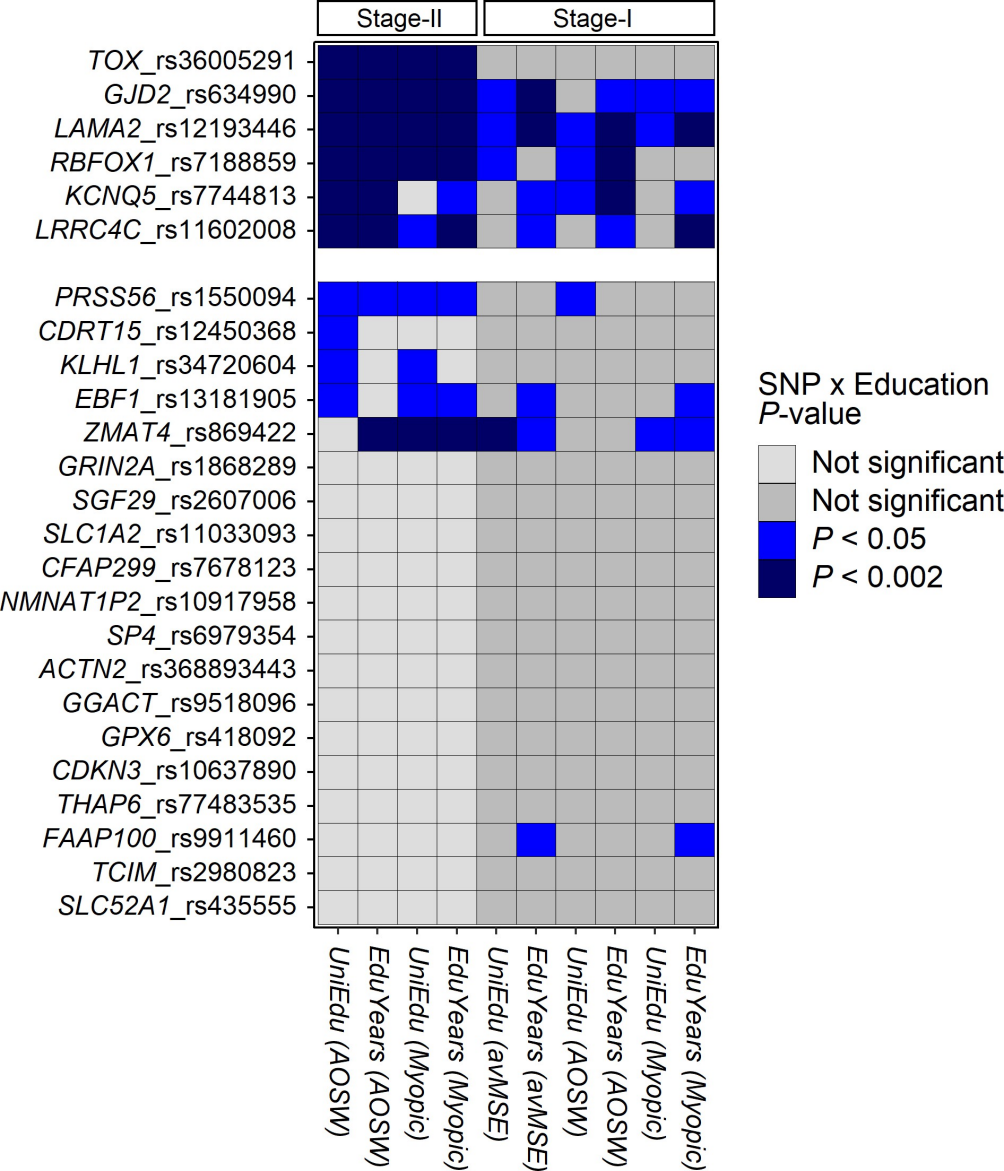
Summary of the evidence for SNP genotype-by-education interactions contributing to myopia development. Each tile indicates the statistical evidence (p-value) for a test of GxE interaction. Genetic variants are listed by row on the y-axis. Tests for different phenotypes (*avMSE*, *AOSW* or *Myopic*) and different indices of education (*UniEdu* or *EduYears*) are listed by column on the x-axis. Note that all GxE tests in the Stage-II sample will be correlated, all GxE tests in the Stage-I sample will be correlated, but that the tests in the Stage-II sample are independent of those in the Stage-I sample.

Given prior work linking education and myopia, we examined if any of the 25 vQTL identified in the 2-step screening strategy displayed genotype-by-education interaction effects. Obtaining a University degree was taken as an index of relatively high educational intensity during childhood: the validity of this index of educational exposure is discussed in Box B of S1 Text. The validity of *AOSW* as a surrogate phenotype for refractive error when testing for gene-by-education interaction effects is discussed in Box C in S1 Text. The GxE interaction test results are presented in Figs 2 and 3, Tables 3 and S2. Six variants, nearby the genes *TOX*, *GJD2*, *LAMA2*, *RBFOX1*, *KCNQ5* and *LRRC4C*, had evidence of a genotype × *UniEdu* interaction in the Stage-II sample after accounting for multiple testing (*P* < 0.002; Bonferroni correction for 25 tests). Similar results were obtained when considering the interaction between genotype × *EduYears* (the age of completing full-time education) (*P* < 0.002 for all 6 variants; S2 Table). For all 6 variants, additional years spent in education was associated with an increased impact of the SNP risk allele, consistent with education compounding genetic predisposition to myopia (Fig 2 and S2 Table).

**Table 3:** Genotype-by-*UniEdu* (GxE) interaction tests for *AOSW* in the Stage-II sample and for *avMSE* in the Stage-I sample. Results shown for the 25 variants with evidence of variance heterogeneity for *avMSE* in the Stage-I sample. Variants are ranked by p-value for the GxE interaction test in the Stage-II sample. The effect allele is the allele associated with a more myopic refractive error in a linear regression test for the marginal effect of the variant.

Variant	Nearest gene	CHR	POS	EA	NEA	FreqEA	SNP x *UniEdu* interaction for *AOSW*			SNP x *UniEdu* interaction for *avMSE*		
							BETA	95% C.I.	*P*-value	BETA	95% C.I.	*P*-value
rs36005291	*TOX*	8	60179048	CA	C	0.655	-0.630	(-0.839 to -0.422)	3.21E-09*	-0.024	(-0.078 to 0.029)	3.78E-01
rs634990	*GJD2*	15	35006073	C	T	0.492	-0.569	(-0.765 to -0.373)	1.32E-08*	-0.071	(-0.121 to -0.021)	5.63E-03
rs12193446	*LAMA2*	6	129820038	A	G	0.904	-0.887	(-1.219 to -0.554)	1.74E-07*	-0.130	(-0.215 to -0.044)	2.91E-03
rs7188859	*RBFOX1*	16	7460426	C	T	0.365	-0.417	(-0.623 to -0.211)	7.49E-05*	-0.067	(-0.120 to -0.014)	1.38E-02
rs7744813	*KCNQ5*	6	73643289	A	C	0.588	-0.375	(-0.577 to -0.173)	2.76E-04*	-0.038	(-0.090 to 0.014)	1.49E-01
rs11602008	*LRRC4C*	11	40149305	T	A	0.17	-0.477	(-0.739 to -0.214)	3.68E-04*	-0.049	(-0.117 to 0.019)	1.54E-01
rs1550094	*PRSS56*	2	233385396	G	A	0.304	-0.290	(-0.503 to -0.077)	7.69E-03	-0.046	(-0.100 to 0.009)	1.02E-01
rs12450368	*CDRT15*	17	14138507	C	T	0.409	-0.236	(-0.448 to -0.024)	2.94E-02	-0.015	(-0.070 to 0.039)	5.86E-01
rs34720604	*KLHL1*	13	70190188	T	TA	0.729	0.245	(0.023 to 0.467)	3.08E-02	-0.006	(-0.063 to 0.052)	8.50E-01
rs13181905	*EBF1*	5	158492279	A	C	0.436	-0.220	(-0.421 to -0.019)	3.21E-02	-0.032	(-0.083 to 0.020)	2.31E-01
rs869422	*ZMAT4*	8	40723970	A	G	0.794	-0.217	(-0.460 to 0.026)	8.03E-02	-0.100	(-0.163 to -0.038)	1.65E-03
rs1868289	*GRIN2A*	16	10215813	T	G	0.678	-0.175	(-0.386 to 0.036)	1.04E-01	-0.026	(-0.080 to 0.029)	3.56E-01
rs2607006	*SGF29*	16	28576017	C	T	0.414	-0.153	(-0.359 to 0.053)	1.46E-01	-0.025	(-0.078 to 0.027)	3.48E-01
rs11033093	*SLC1A2*	11	35381495	G	C	0.103	-0.232	(-0.555 to 0.091)	1.59E-01	0.027	(-0.056 to 0.110)	5.21E-01
rs7678123	*CFAP299*	4	81372405	C	G	0.284	-0.152	(-0.370 to 0.066)	1.73E-01	-0.017	(-0.074 to 0.039)	5.42E-01
rs10917958	*NMNAT1P2*	1	164214985	T	C	0.234	-0.131	(-0.363 to 0.101)	2.68E-01	0.021	(-0.038 to 0.081)	4.85E-01
rs6979354	*SP4*	7	21417963	T	C	0.555	0.092	(-0.111 to 0.294)	3.76E-01	-0.030	(-0.082 to 0.022)	2.54E-01
rs368893443	*ACTN2*	13	100690003	AAGAG	A	0.454	0.087	(-0.111 to 0.285)	3.91E-01	-0.006	(-0.057 to 0.045)	8.27E-01
rs9518096	*GGACT*	13	101223109	C	T	0.481	0.073	(-0.124 to 0.270)	4.66E-01	-0.012	(-0.063 to 0.038)	6.30E-01
rs418092	*GPX6*	6	28533946	T	C	0.333	0.062	(-0.147 to 0.270)	5.63E-01	0.012	(-0.042 to 0.065)	6.72E-01
rs10637890	*CDKN3*	14	54779523	T	TCT	0.375	-0.058	(-0.263 to 0.147)	5.81E-01	-0.013	(-0.066 to 0.040)	6.25E-01
rs77483535	*THAP6*	4	76312573	T	C	0.302	0.059	(-0.163 to 0.281)	6.01E-01	-0.008	(-0.064 to 0.049)	7.94E-01
rs9911460	*FAAP100*	17	79538841	T	A	0.477	-0.036	(-0.234 to 0.161)	7.19E-01	-0.044	(-0.095 to 0.006)	8.55E-02
rs2980823	*TCIM*	8	40148843	G	T	0.582	0.026	(-0.174 to 0.226)	8.00E-01	0.029	(-0.022 to 0.081)	2.67E-01
rs435555	*SLC52A1*	17	4947305	T	C	0.556	-0.023	(-0.222 to 0.175)	8.18E-01	-0.022	(-0.072 to 0.029)	4.03E-01

*GxE interaction test *P* < 0.002 in the Stage-II sample.

Abbreviations: CHR = Chromosome; POS = Genomic position for GRCh37; EA = Effect allele; NEA = Non-effect allele; FreqEA = Allele frequency of effect allele; BETA = Regression coefficient for the interaction term (units for outcome *AOSW*: years per copy of the risk allele in those with vs. without a University degree; units for outcome *avMSE*: D per copy of the risk allele in those with vs. without a University degree); CI = Confidence interval.

The effect size of the genotype × *UniEdu* interaction was highly correlated between the Stage-I and Stage-II samples for the 25 variants (Spearman ρ = 0.71, *P* = 9.5 x 10^−5^). Full results of tests for genotype × *UniEdu* and genotype × *EduYears* interactions for the phenotypes *avMSE*, *AOSW* and *Myopic* in the Stage-I and Stage-II samples are presented in Figs 3 and B in S1 Text and S2 Table. For 5 of the 6 lead GxE variants (all those except rs36005291 nearby the *TOX* gene) there was independent evidence for a SNP × *UniEdu* and/or SNP × *EduYears* interaction in the Stage-I cohort (Table 3 and Fig 3).

Statistical evidence for the presence of a GxE interaction can be heavily dependent on the choice of measurement scale of the phenotype [20,21]. Therefore, as a sensitivity analysis, we examined if the above interactions were specific to the chosen measurement scale (an additive-scale) by performing logistic regression analyses (on the log-odds scale) in the Stage-II sample for the ‘myopia case-control status’ phenotype. Of the 6 variants examined, 5 retained evidence of a genotype × *UniEdu* interaction (*P* < 0.05 for all variants except *KCNQ5* variant rs7744813) and all 6 retained evidence of a genotype × *EduYears* interaction (*P* < 0.05) when tested for an association with myopia case-control status (Figs 2 and 3 and S2 Table). Additional sensitivity analyses confirmed that the results were robust to whether statistical adjustment for *UniEdu* was or was not performed [19] in Step 1 and Step 2 (Box D in S1 Text).

For non-normally distributed traits such as refractive error, SNPs with marginal effects may also be associated with the trait variance (a ‘mean-variance relationship’). Heteroskedastic linear mixed models (HLMMs) have been developed to detect variance heterogeneity after accounting for such a relationship [22–24]. Pozarickij et al. [25] reported a HLMM-based GWAS analysis for refractive error in the UK Biobank sample, which yielded 14 independent variants with genome-wide significant evidence of variance heterogeneity (*P* < 5 x 10^−8^; see Table 3.4 of [25] for details). All 6 of the variants with significant genotype × education interaction effects in the Stage-II sample were among the 14 variants identified using HLMM by Pozarickij et al. (or were in high LD with one of these 14 variants).

**Fig 4:**
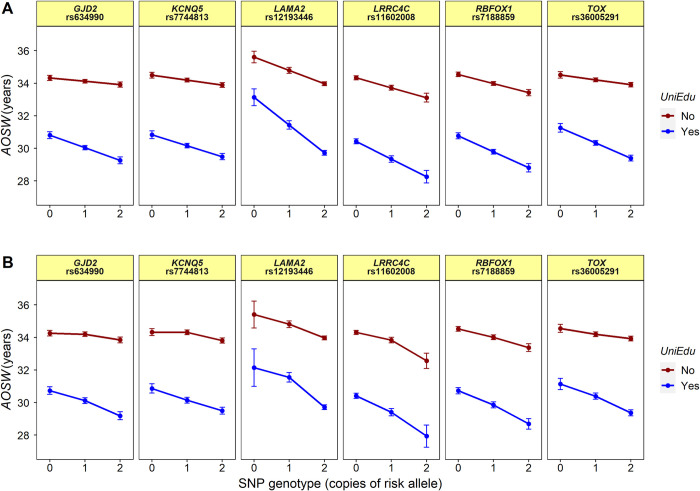
Modeling genotype-by-education interactions when allowing for dominance effects. The plots illustrate the relationship between SNP genotype, exposure to University education (*UniEdu*) and the *AOSW* phenotype in the Stage-II sample when SNP genotypes were coded as either numeric (i.e. assuming additive effects; panel A) or categorical (allowing for dominant or recessive effects; panel B).

The above tests for genotype-by-education interaction effects were performed under the assumption that SNPs had additive effects. This meant, for example, that the change in phenotype expected for individuals with a University education would be shifted by an amount *β*_3_ in those with 1 copy of the SNP effect allele and by 2*β*_3_ in those with 2 copies of the effect allele (see *Eq* 1 and *Eq* 2 in *Methods*). We previously observed that the *LAMA2* lead variant effect allele may act recessively rather than additively [26]. Since dominant or recessive genetic variants tested using an additive model can give rise to spurious evidence of a GxE interaction [24], we explored if the genotype-by-*UniEdu* interactions of the *TOX*, *GJD2*, *LAMA2*, *RBFOX1*, *KCNQ5* and *LRRC4C* variants were robust to the choice of model specification. As shown in Table 4 and Fig 4, the most parsimonious model was an additive model for all of the variants, except for *LAMA2*, which was better fit by a recessive model. However, the evidence for a genotype-by-*UniEdu* interaction was still evident for the *LAMA2* variant under a recessive model, suggesting the evidence for interaction effects did not arise as a result of genetic model misspecification.

**Table 4:** Tests for genotype-by-education interaction when allowing for dominance effects. Linear regression analyses were performed for the *AOSW* phenotype in the Stage-II sample for genotype-by-*UniEdu* interactions when the SNP effect allele was coded as additive (0, 1 2), fully dominant (0, 1, 1) or fully recessive (0, 0, 1). The effect allele is the allele associated with a more myopic refractive error in a linear regression test for an additive marginal effect of the variant.

Variant	Nearest gene	EA	NEA	Additive model GxE *P*-value	Dominant model GxE *P*-value	Recessive model GxE *P*-value	Additive model -2*logLik	Dominant model -2*logLik	Recessive model -2*logLik
rs36005291	*TOX*	CA	C	3.21E-09*	6.11E-04	6.42E-09	2072926*	2073001	2072947
rs634990	*GJD2*	C	T	1.32E-08*	3.76E-06	3.25E-06	2119565*	2119614	2119580
rs12193446	*LAMA2*	A	G	1.74E-07	2.55E-01	6.30E-08*	2119444	2119644	2119443*
rs7188859	*RBFOX1*	C	T	7.49E-05*	1.39E-03	9.04E-04	2061642**	2061706	2061721
rs7744813	*KCNQ5*	A	C	2.76E-04*	5.66E-05	3.16E-02	2050158*	2050203	2050168
rs11602008	*LRRC4C*	T	A	3.68E-04*	3.36E-04	1.77E-01	2101306*	2101339	2101385

* Indicates the result for the most parsimonious model.

Abbreviations: EA = Effect allele; NEA = Non-effect allele; GxE = Gene-by-Environment interaction term in model; logLik = log likelihood.

### Exclusion of gene-environment correlation

SNPs directly associated with both educational attainment and refractive error could potentially yield spurious GxE interaction test findings, via ‘gene-environment correlation’ (rGE) [21,27]. Dudbridge and Fletcher [27] describe examples of the conditions under which rGE can lead to significant GxE interaction effects despite the genetic marker used in the GxE test having no causal effect on the outcome trait, while Dick et al. [21] discuss approaches for testing GxE interactions in the presence of rGE. As shown in Table 5, the only variant with evidence of an rGE effect was *LRRC4C* variant, rs11602008. This SNP had a weak association with *UniEdu* (OR = 1.017; *P* = 0.034). Thus, the data did not support rGE effects as an explanation for the interaction effects observed in the current study.

**Table 5:** Gene-environmental correlation (rGE) between genetic variants and University education in the Stage-II sample. SNPs were tested using logistic regression. The effect allele is the allele associated with a more myopic refractive error in a linear regression test for the marginal effect of the variant. The frequency of the effect allele is reported in participants who did or did not attend University (*UniEdu* = *Yes*/*No*).

Variant	Nearest gene	EA	NEA	FreqEA *UniEdu* = *Yes*	FreqEA *UniEdu* = *No*	Odds ratio	95% C.I.	*P*-value
rs36005291	*TOX*	CA	C	0.656	0.654	0.992	(0.979 to 1.004)	0.195
rs634990	*GJD2*	C	T	0.492	0.492	1.002	(0.990 to 1.015)	0.691
rs12193446	*LAMA2*	A	G	0.905	0.904	0.991	(0.971 to 1.012)	0.392
rs7188859	*RBFOX1*	C	T	0.363	0.366	0.988	(0.976 to 1.001)	0.067
rs7744813	*KCNQ5*	A	C	0.587	0.588	1.003	(0.991 to 1.016)	0.606
rs11602008	*LRRC4C*	T	A	0.171	0.169	1.017	(1.001 to 1.034)	0.034

Abbreviations: EA = Effect allele; NEA = Non-effect allele; FreqEA = Allele frequency of effect allele; C.I. = confidence interval.

### Tests for gene-gene interaction

Variance heterogeneity is an expected feature for genetic variants involved in GxG interactions, as well as those involved in GxE interactions [16]. Therefore, each of the 300 possible pairs of SNPs from amongst the 25 SNPs with evidence of variance heterogeneity was tested for a genotype-by-genotype interaction with the *avMSE* phenotype in the Stage-I sample and with the *AOSW* phenotype in the Stage-II sample. Only one pair of SNPs had evidence of a GxG interaction after accounting for multiple testing (*P* < 0.05/300). This was the interaction between rs12193446 × rs7744813, nearby the *LAMA2* and *KCNQ5* genes, which was strongly associated with *AOSW* in the Stage-II sample (*P* = 6.31 x 10^−5^; Fig C in S1 Text). However, there was no evidence of an interaction associated with *avMSE* for this pair of SNPs in the Stage-I sample (*P* = 0.97).

## Discussion

This work identified 25 genetic variants associated with a difference in refractive error *variance* across genotypes, after accounting for multiple testing. Of these 25 variants, 19 also exhibited evidence of variance heterogeneity in association with *AOSW* in an independent sample, confirming them as bona fide vQTL. Consistent with the expectation that refractive error vQTL would be enriched for variants with GxE interaction effects, 6 genetic variants had evidence of genotype-by-education interaction effects associated with *AOSW* in the Stage-II sample, after accounting for multiple testing. For 5 of the 6 variants, which were located nearby the genes *GJD2*, *LAMA2*, *RBFOX1*, *KCNQ5* and *LRRC4C*, there was independent evidence of a genotype-by-education interaction associated with refractive error in the Stage-I sample. For all variants, greater exposure to education was associated with an increased effect size for the myopia-predisposing risk allele, consistent with evidence that education is a causal risk factor for myopia [10–12,28]. Variants in 2 of the 5 genes with robust evidence of a GxE interaction, *GJD2* and *RBFOX1*, have previously been reported to be involved in gene-by-education interactions influencing myopia [14]. The 3 remaining genes identified here (*LAMA2*, *KCNQ5* and *LRRC4C*) are novel GxE interaction discoveries. The 25 genetic variants associated with refractive error variance are also promising candidates for involvement GxG interactions. However, the evidence for GxG interactions in the current samples was limited, with a single pair of variants displaying evidence of an interaction in the larger Stage-II sample but not in the smaller Stage-I sample (Fig C in S1 Text).

The role of GxE interactions in myopia has recently been reviewed [29]. The current work brings the number of independently replicated gene-by-education interactions for myopia to 3 (*ZMAT4*, *GJD2* and *RBFOX1*). In a meta-analysis of five studies from Singapore, Fan et al. [14] reported that *GJD2* variant rs524952 and *RBFOX1* variant rs17648524 (both in perfect LD with the variants in *GJD2* and *RBFOX1* studied here) were associated with a greater risk of myopia in individuals from a high vs. low education stratum. The gene-by-education interaction effect size for these two variants was larger in the East Asian cohorts studied by Fan et al. [14] than the current UK-based sample (*β*_*G*×*E*_ ≈ -0.25 D in the Fan et al. study vs. *β*_*G*×*E*_ ≈ -0.07 D in the current study), consistent with prior findings that GxE effects on myopia are larger in East Asians [15]. Several other genes have also been associated with gene-by-education interactions affecting refractive error: *TRPM1*, *MMP2*, *SHISA6*, *DNAH9*, *ZMAT4*, *SFRP1*, *AREG*, *GABRR1*, *PDE10A*, *APLP2*, *DLX1*, *BICC1* and *A2BP1* [5,14,15,30–33]. Of these genes, only *ZMAT4* was located nearby the 25 variants selected by our 2-step screening protocol. In the current analysis, rs869422 in *ZMAT4* displayed evidence of an interaction with *UniEdu* and *EduYears* in the Stage-I sample (Fig 3; *UniEdu*: *β*_*G*×*E*_ = -0.10 D, *P* = 1.65 x 10^−3^; *EduYears*: *β*_*G*×*E*_ = -0.17 D, *P* = 3.77 x 10^−3^) and with *EduYears* in the Stage-II sample (*β*_*G*×*E*_ = -0.07 years, *P* = 1.65 x 10^−3^), however the genotype × *UniEdu* interaction test in the Stage-II sample was non-significant (*P* = 0.08). This illustrates the challenge of detecting GxE interaction effects in myopia development.

*GJD2* is one of the most intensively studied myopia-susceptibility genes [34–36]. *GJD2* encodes the neuronal gap junction protein connexin-36 (Cx36), which is thought to play a role in ON-bipolar cell signaling and cone-driven OFF pathways in the retina [35,37,38]. Loss-of-function mutations in connexin-36 in a Zebrafish model inhibit eye growth and diminish the electroretiniogram B-wave amplitude [35]. *RBFOX1* encodes a member of the Fox-1 family, which regulate tissue-specific alternative splicing. As well as refractive error [39], *RBFOX1* polymorphisms are associated with blood pressure [40], allergy [41] and lung cancer [42]. Presently, it is unclear how Fox-1 proteins confer susceptibility to myopia. *KCNQ5* encodes the ‘potassium voltage-gated channel subfamily Q member 5’ protein, which forms M-type potassium channels in the inner and outer plexiform layers, rod and cone photoreceptors, and the RPE of the primate retina [43]. In a guinea pig myopia model, retinal *Kcnq5* gene and protein expression were down-regulated [44]. In adult twins, *KCNQ5* variant rs2840795 was associated with electroretiniogram B-wave responses [45]. However, rs2840795 is in weak LD (r^2^ = 0.03) with the strongest myopia-predisposing variants in the region, rs7744813. *LAMA2* encodes the alpha-2 laminin subunit. Laminin is a major component of basement membranes that has multiple roles, including the attachment of cells to the matrix [46]. *LAMA2* variant rs12193446 is associated with refractive error early in life and then progressively through childhood [47], consistent with children’s duration of exposure to education. *LRRC4C* encodes ‘leucine rich repeat containing 4C’, also known as netrin-G ligand-1 (NGL1). Netrins are axon guidance molecules related to laminin, highly expressed in the central nervous system during embryological development, which attract or repel axons to guide them to their correct target. Netrin-G1 is an atypical netrin, being diffusible rather than tethered to the cell membrane. NGL1 binds intracellularly with PSD-95, a postsynaptic scaffolding protein, and extracellularly with netrin-G1. This protein complex is thought to control the development of distinct populations of neuronal synapses and neural circuits [48]. Other *LRRC4C* variants are associated with developmental disorders such as autism [49]. *Lrrc4c* knockout mice display hyperactivity and anxiety-like behaviors [48,49].

We used Levene’s median test to assess variance heterogeneity, since previous work has demonstrated this test maintains good control of the type-1 error rate for non-normally distributed phenotypes, of which *avMSE* and *AOSW* are examples [17,50]. One potential limitation of Levene’s test is that it cannot incorporate covariates, however we addressed this issue by first regressing the phenotype on covariates and then applying Levene’s test to the residuals (although we note that this approach may be biased for variants that are correlated with covariates [51]). Moreover, for any trait with a non-normal distribution, there will be a relationship between the mean and the variance of the trait [24]. Genetic variants associated with the variance of a trait purely as a consequence of such a general mean-variance relationship are unlikely to provide mechanistic insight into GxE interactions. Hence, several statistical tests designed to detect residual variance heterogeneity (‘dispersion’) not accounted for by a general mean-variance relationship have been developed [22–24]. Unfortunately, these tests can produce a high false-positive rate for non-normally distributed phenotypes [18] and it has been shown that trait transformation invariably leads to false positives in simulations where SNPs only affect the mean of the phenotype, regardless of the variance heterogeneity test used [18]. Together, these findings suggest there is no ideal method for detecting vQTL for non-normally distributed phenotypes. Nevertheless, in preliminary work [25] in which we applied the heteroskedastic linear model described by Young et al. [24], the 6 variants nearby *TOX*, *GJD2*, *LAMA2*, *RBFOX1*, *KCNQ5* and *LRRC4C* demonstrated genome-wide significant dispersion effects, i.e. variance heterogeneity not accounted for by a general mean-variance relationship.

One route through which education may influence refractive error is increased near work and accommodation. Children typically show a small deficit (‘lag’) of accommodation, compared to the level required to focus precisely. The accommodative lag theory of myopia development proposes the hyperopic defocus on the retina accompanying accommodative lag acts as a physiological signal for increased axial elongation [52]. However, evidence for the accommodative lag theory is mixed [52–55] and other aspects of the near visual environment have been put forward to explain the link between near work and myopia [56–58]. Educational activities typically take place indoors. Since insufficient time outdoors is an established risk factor for myopia [9,59,60], exposure to education may also serve as a proxy for time spent indoors.

Strengths of the current study include the large sample size, the use of highly standardised methods for assessing phenotypes and risk factors, and the exclusion of gene-environment correlation effects. Limitations of the study were that autorefraction was not carried out on all UK Biobank participants, which reduced the sample size for the Stage-I sample, and that–in order to maintain high statistical power–attention was focused on a set of 25 variants enriched for myopia-associated GxE interaction effects. This approach would have excluded variants that did not have both a marginal effect and a variance heterogeneity association with refractive error, such as variants with ‘cross-over’ type GxE interaction effects in which the genetic effect cancels out under different environmental conditions (right panel of Fig 1). A further limitation was that the participants were adults 50–60 years of age from the UK. There has been a steady increase in years spent in education in many parts of the world over the past few decades. Therefore, studying gene-environment interactions in individuals who have more recently undergone their education or those in countries with more intensive education systems may be beneficial in detecting the full impact of GxE interactions in contemporary populations.

In summary, we identified 25 genetic variants with evidence of variance heterogeneity in their association with refractive error. Nineteen of the 25 variants also demonstrated evidence of variance heterogeneity for *AOSW* in an independent sample. These vQTLs are strong candidates for having either GxE or GxG interaction effects and, indeed, genetic variants located near the *GJD2*, *LAMA2*, *RBFOX1*, *KCNQ5* and *LRRC4C* genes were associated with a progressively increasing risk of myopia as the number of years of schooling rose. Three of these gene-education interaction findings were novel (those implicating *LAMA2*, *KCNQ5* and *LRRC4C*), while the remaining two supported interactions identified previously in cohorts from East Asia. More research is needed to understand the biological pathways through which these 5 variants act, and how they interact with the effect of near work, intensive education, or insufficient time outdoors (for which education may be a proxy).

## Methods

### Ethics statement

The UK Biobank study had ethical approval from the United Kingdom National Health Service (NHS) Research Ethics Committee (Reference: 11/NW/0382). Signed and informed consent was obtained from all of the participants.

Full details of the methods are provided in S1 Text. Unless stated otherwise, all tests were performed in R [61].

### Participants, phenotype and environmental variables

Analyses were performed on data from participants in UK Biobank, a prospective study examining the health and wellbeing of 500,000 adults aged 40–70 years-old living in the United Kingdom [62]. Baseline assessment visits occurred between 2006–2010. Information about education level and age of completing full-time education were obtained from a questionnaire. The binary variable *UniEdu* was used to indicate whether individuals had a University or college degree. The variable *EduYears* was used to classify the age at which the participants completed their full-time education [11]. Participants self-reported their age-of-onset of spectacle wear (*AOSW*) [63], which was coded as a continuous phenotype. An ophthalmic assessment was introduced into UK Biobank only towards the later stages of recruitment; approximately 23% of participants were assessed [64]. The refractive error phenotype (*avMSE*) was calculated as the average spherical equivalent of both eyes from non-cycloplegic autorefraction. DNA extraction, genotyping and imputation were performed as described [65]. A ‘Stage-I’ sample of unrelated participants of European ancestry (N = 88,334) with information for *avMSE*, *UniEdu* and *EduYears* was selected. Participants were classified as myopic if they had an *avMSE* ≤ -0.50 D [66]. A Stage-II sample of European-ancestry participants (N = 252,838) was selected who were unrelated to each other, unrelated to any person in the Stage-I sample, and who had information available for *AOSW*, *UniEdu* and *EduYears*. (Under this classification scheme, participants who did not wear spectacles, and therefore did not report an AOSW, were not included in the Stage-II sample). Participants in the Stage-II sample were classified as myopic if they had an *AOSW* greater than 5 years and less than or equal to 25 years [67]. There were too few participants of non-European ancestry to study GxE interactions in other ancestry groups.

### Two-step screening strategy for identifying putative GxE interaction variants

Step 1 was a standard GWAS for the phenotype *avMSE* in the Stage-I sample (Fig 1A), implemented with BOLT-LMM [68]. Sex, age, age-squared, a binary indicator of the genotype array (UK BiLEVE Axiom or UK Biobank Axiom array) and the first 10 ancestry principal components (PCs) were included as covariates. Approximately 7 million imputed genetic variants with a minor allele frequency >5% were tested. Independently associated SNPs were selected by clumping with PLINK [69]. SNPs associated with *avMSE* at the lenient threshold of *P* < 1 x 10^−4^ were taken forward to Step 2, which was a variance heterogeneity analysis for the phenotype *avMSE* in the Stage-I sample (Fig 1B) using Levene’s median test, implemented with OSCA [17] and following the approach recommended by Zhang et al. [19]. In total, 956 SNPs were taken forward to Step 2. A Bonferroni correction for multiple comparisons was applied (α = 0.05/956 = 5.23 × 10^−5^). In the absence, worldwide, of a study cohort of comparable size to UK Biobank with data available for *avMSE* and high-density genotypes, confirmation of the variance heterogeneity findings in the Stage-I sample were sought by performing Levene’s test for the trait *AOSW* in the Stage-II sample. Consideration of the validity of *AOSW* as a surrogate phenotype for refractive error when testing for gene-by-education interaction effects is discussed in Box C in S1 Text.

### Gene-environment interaction tests

For each of the 25 SNPs identified by the 2-step screening protocol, linear regression models with an interaction term were fitted in the Stage-I sample (*Eq* 1) and the Stage-II sample (*Eq* 2):

$$avMSE=\beta_{0}+\beta_{1}SNP+\beta_{2}UniEdu+\beta_{3}SNP\times UniEdu+\gamma C+\varepsilon$$ (1)

$$AOSW=\delta_{0}+\delta_{1}SNP+\delta_{2}UniEdu+\delta_{3}SNP\times UniEdu+\gamma C+\pi$$ (2)

Where, *avMSE* and *AOSW* are *n* × 1 vectors of refractive error and age-of-onset of spectacle wear values in the *n* participants in the Stage-I sample and Stage-II sample, respectively. *SNP* is a *n* × 1 vector of genotype counts (0, 1 or 2), *UniEdu* is a *n* × 1 vector binary (0,1) variable for University level education, *C* is a *n* × *k* matrix of covariates (age, age-squared, genotyping array, and the first 10 ancestry PCs), γ is a 1 × *k* vector of regression coefficients, and ε and *π* are residuals assumed to be normally distributed. *β*_0_ is an intercept, while *β*_1_, *β*_2_ and *β*_3_ are the regression coefficients for the main effect for the SNP, the main effect for *UniEdu* and the SNP × *UniEdu* interaction effect, respectively (likewise, for *δ*_0_, *δ*_1_, *δ*_2_ and *δ*_3_). Linear regression models of the same form, but with robust standard errors, were also fitted to test for genotype-by-*EduYears* interactions associated with either *avMSE* or *AOSW*.

Analogously, logistic regression models of the same form to *Eq* 1 and *Eq* 2 above were applied to test for genotype-by-education interaction effects associated with the outcome variable *Myopic* (1 = myopic, 0 = non-myopic). Simulations were used to confirm that the type-1 error rate of the genotype-by-education test for the *AOSW* phenotype was well-controlled under the test conditions, despite the non-normal distribution of *AOSW* and in the presence of GxE interactions not involving education (Box A in S1 Text).

### Gene-gene interaction tests

SNP × SNP interaction tests were performed to examine if any pair of SNPs from amongst the 25 SNPs identified using the 2-step screening strategy had evidence of a genotype × genotype interaction. A linear regression model with an interaction term was fitted for each pair of variants in turn, as follows:

$$avMSE=\beta_{0}+\beta_{1}SNP1+\beta_{2}SNP2+\beta_{3}SNP1\times SNP2+\gamma C+\varepsilon$$ (3)

$$AOSW=\delta_{0}+\delta_{1}SNP1+\delta_{2}SNP2+\delta_{3}SNP1\times SNP2+\gamma C+\pi$$ (4)

Where terms are defined as above. The *avMSE* phenotype was tested in the Stage-I sample and the *AOSW* phenotype was tested in the Stage-II sample. A Bonferroni correction for multiple comparisons was applied to identify *δ*_3_ or *β*_3_ terms showing evidence of association, using an alpha value of α = 0.05/300 = 0.00017 (accounting for a total of 25×25 / 2 tests).

### Gene-environment correlation tests

To test for gene-environment correlation, the following logistic regression model was fitted:

$$logit\;P(UniEdu=1|SNP,C)=\omega_{0}+\omega_{1}SNP+\gamma C+\varepsilon$$ (5)

Where, *ω*_0_ is an intercept and *ω*_1_ quantifies the association between the SNP genotype and having a University degree.

### Sensitivity analyses

We carried out simulations to assess the type-1 error rate of Levene’s median test for variance heterogeneity with the *avMSE* phenotype in the Stage-I sample and with the *AOSW* phenotype in the Stage-II sample, as well as the type-1 error rate of linear regression when testing for a SNP × *UniEdu* interaction with the *AOSW* phenotype in the Stage-II sample.

## Supporting information

S1 Table Summary statistics for the 956 SNPs independently associated with refractive error in the GWAS for *avMSE* in the Stage-I sample. (XLSX)

S2 Table Marginal effect genetic association tests and genotype-by-*UniEdu* and genotype-by-*EduYears* interaction tests for refractive error-related phenotypes (*avMSE*, *AOSW*, and *Myopic*) in the Stage-I sample and Stage-II sample. (XLSX)

S1 Text **Box A in S1 Text**. Type-1 error rates. **Box B in S1 Text**. Validity of University education as an index of educational intensity. **Box C in S1 Text**. Validity of age-of-spectacles-wear (AOSW) as a surrogate for refractive error (avMSE) in GxE interaction tests. **Box D in S1 Text**. Comparison of results of sensitivity analyses. **Box E in S1 Text**. Supplementary Methods. **Fig A in S1 Text**. Manhattan plot of the results from the GWAS for refractive error (avMSE) in the Stage-I sample. **Fig B in S1 Text**. SNP genotype by University education (GxE) interactions associated with age-of-onset of spectacle wear (AOSW). **Fig C in S1 Text**. Summary of the evidence for SNP × SNP interactions contributing to myopia development.
(PDF)
