# Supplementary material for: Education interacts with genetic variants near GJD2, RBFOX1, LAMA2, KCNQ5 and LRRC4C to confer susceptibility to myopia
Source: PLoS Genet. 2022 Nov 17;18(11):e1010478. doi: 10.1371/journal.pgen.1010478 (PMC9671369; doi:10.1371/journal.pgen.1010478)
Supplement: S1 Text — Box A in S1 Text. Type-1 error rates. Box B in S1 Text. Validity of University education as an index of educational intensity. Box C in S1 Text. Validity of age-of-spectacles-wear (AOSW) as a surrogate for refractive error (avMSE) in GxE interaction tests. Box D in S1 Text. Comparison of results of sensitivity analyses. Box E in S1 Text. Supplementary Methods. Fig A in S1 Text. Manhattan plot of the results from the GWAS for refractive error (avMSE) in the Stage-I sample. Fig B in S1 Text. SNP genotype by University education (GxE) interactions associated with age-of-onset of spectacle wear (AOSW). Fig C in S1 Text. Summary of the evidence for SNP × SNP interactions contributing to myopia development. (PDF) [file pgen.1010478.s003.pdf]

Education interacts with genetic variants near *GJD2, RBFOX1, LAMA2, KCNQ5* and *LRRC4C* to confer susceptibility to myopia

S1 Text

Contents

Box A. Type-1 error rates. 2

Box B. Validity of University education as an index of educational intensity 4

Box C. Validity of age-of-spectacles-wear (*AOSW*) as a surrogate for refractive error (*avMSE*) in GxE interaction tests 5

Box D. Comparison of results of sensitivity analyses. 6

Box E. Supplementary Methods 7

Fig A. Manhattan plot of the results from the GWAS for refractive error (*avMSE*) in the Stage-I sample. 10

Fig B. SNP genotype-by-University education (GxE) interactions associated with age-of-onset of spectacle wear (*AOSW*). 11

Fig C. Summary of the evidence for SNP × SNP interactions contributing to myopia development. 12

Supplementary References 13

Box A. Type-1 error rates.

To preserve the structure of the original dataset, such as the existing relationship between *UniEdu* and *avMSE* or *AOSW*, we simulated SNPs directly in the Stage-I and Stage-II samples.

(a) Type-1 error rate of Levene's median test when testing for variance heterogeneity with the *avMSE* phenotype in the Stage-I sample.
We simulated a biallelic SNP *S* with MAF in the range 0.05–0.50. We took the original *avMSE* phenotype and added a small value to create a new phenotype *Y* such that *S* had an association with *Y* that explained 1% of the variance in *Y*, but *Y* retained an almost identical distribution to *avMSE*. We regressed *Y* on the covariates sex, age, age-squared, genotyping array, and the first 10 PCs and then tested the residuals for variance heterogeneity between the (0, 1 or 2) genotype classes of *S* using Levene's median test. Thus, *S* had a marginal effect but no GxE interaction effect, therefore this provided an assessment of the type-1 error rate for the variance heterogeneity analysis. We carried out the simulation with 5,000,000 replicates.

(b) Type-1 error rate of Levene's median test when testing for variance heterogeneity with the *AOSW* phenotype in the Stage-II sample.
As above, we simulated a biallelic SNP *S* with MAF in the range 0.05–0.50. We modified the original *AOSW* phenotype to create a new phenotype *Y* such that *S* had an association with *Y* that explained 1% of the variance in *Y*, but *Y* retained an almost identical distribution to *AOSW*. We regressed *Y* on the covariates sex, age, age-squared, genotyping array, and the first 10 PCs and then tested the residuals for variance heterogeneity by *S* using Levene's median test. Thus, *S* had a marginal effect but no GxE interaction effect, therefore this provided an assessment of the type-1 error rate for the variance heterogeneity analysis. We carried out the simulation with 5,000,000 replicates.

(c) Type-1 error rate of linear regression when testing for a SNP × *UniEdu* interaction with the *AOSW* phenotype in the Stage-II sample.
We simulated a biallelic SNP *S* with MAF in the range 0.05–0.50 together with a binary variable *E* with the same prevalence as *UniEdu* to represent an independent environmental risk factor. We modified the original *AOSW* phenotype to create a new phenotype *Y* with an almost identical distribution to *AOSW* that had an association with *S*, an association with *E*, and an $S \times E$ interaction effect. The variance in *Y* explained by *S*, *E* and the $S \times E$ interaction were each approximately 1%. We then tested for a $S \times UniEdu$ interaction effect by fitting the model:

$$AOSW = \beta_0 + \beta_1 S + \beta_2 UniEdu + \beta_3 S \times UniEdu + \gamma C + \varepsilon \quad (Eq.\ \text{S1})$$

Thus, *S* had a marginal effect, a GxE interaction effect with *E*, but no GxE interaction effect with *UniEdu*. Therefore *Eq*. S1 provided an assessment of the type-1 error rate for the GxE interaction test. We carried out the simulation with 5,000,000 replicates.

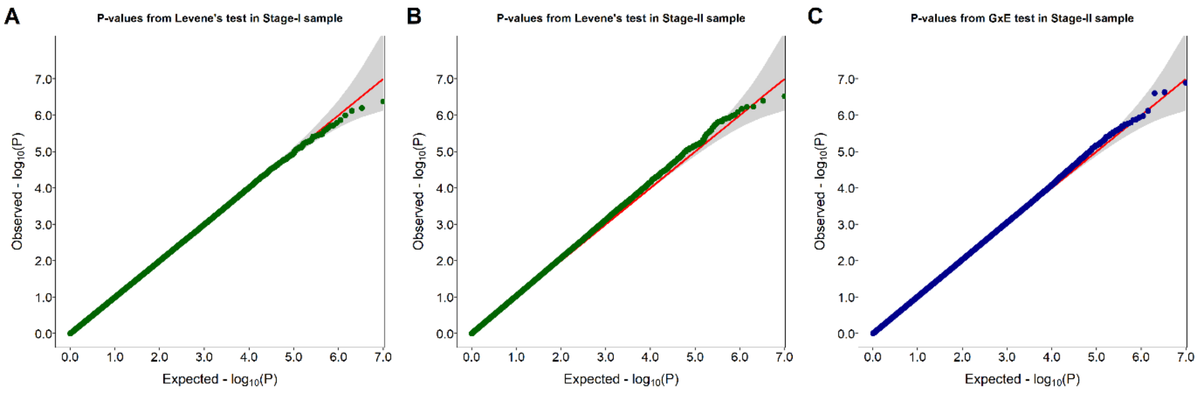

The results are presented in the QQ-plots above. Simulations (a) and (b) suggested appropriate control of the type-1 error rate for Levene's test despite the non-normal distributions of *avMSE* and *AOSW*. Likewise, simulation (c) suggested excellent control of the type-1 error rate when testing for GxE interactions using linear regression despite the highly non-normal distributions of *AOSW*.

Box B. Validity of University education as an index of educational intensity

The presence vs. absence of University education, coded via the binary variable *UniEdu*, served as the primary environmental exposure in this work. *UniEdu* was selected in preference to the discrete variable *EduYears* because the distribution of *EduYears* was highly non-normal and its measurement range was truncated, which may have impacted on the power and type-1 error rate of tests for a GxE interaction.

University education in UK Biobank participants typically began at the age of 18 years-old, which is after the age that myopia usually developed. Hence, the use of *UniEdu* as an index of education attainment could be viewed as paradoxical as regards the time-ordering of exposure and outcome, when testing for a gene-by-education interaction that contributes to myopia development.

However, as elegantly shown by Howe et al. [13], there is evidence that the causal effect of education on *AOSW* (a surrogate for refractive error and myopia) occurs throughout childhood. Thus, in line with Howe et al. [13] and previous GxE interaction studies of myopia [14, 15] we made the assumption that *UniEdu* and *EduYears* capture aspects of educational intensity. Thus, even though a SNP x education interaction may predispose a child to develop myopia and thus require spectacles at an early age, we argue that it is logical to test for a SNP x *UniEdu* interaction effect associated with the outcome *AOSW* under the assumption that attending University serves as a proxy for a relatively high level of *educational intensity throughout childhood*.

Box C. Validity of age-of-spectacles-wear (*AOSW*) as a surrogate for refractive error (*avMSE*) in GxE interaction tests

The strongest evidence that *AOSW* is a valid surrogate for refractive error when testing for GxE interactions is that we observed a high correlation between the SNP × *UniEdu* interaction effect size $\beta_{G\times E}$ for the trait *AOSW* in the Stage-II sample and the SNP × *UniEdu* interaction effect size for the trait *avMSE* in the Stage-I sample (Spearman $\rho = 0.71$, $P = 9.48 \times 10^{-5}$, for the 25 genome-wide significant vQTL variants; Figure 3A). Furthermore, the p-values from Levene's test for *AOSW* in the Stage-II sample and the p-values from Levene's test for *avMSE* in the Stage-I sample were also highly correlated (Spearman $\rho = 0.58$, $P = 0.003$). This correlation implies that sources of variance heterogeneity for the trait *avMSE* can be detected when using *AOSW* as a surrogate trait. More generally, the relationship between *AOSW* and refractive error is strong and direct (for those aged < 40 years-old, refractive error is typically the reason why individuals start wearing glasses) [16-19]. The two traits have a very high genetic correlation ($r_g = -0.97$) [18]). In UK Biobank participants, a polygenic score for *AOSW* and a polygenic score for *avMSE* each explained approximately 7% of the variance in *avMSE* in an independent sample of participants [4]. Finally, there are several precedents for using *AOSW* as a validation phenotype for refractive error [7, 13, 20].

Box D. Comparison of results of sensitivity analyses.

Sensitivity analyses were carried out in which statistical adjustment for the marginal effect of *UniEdu* was or was not performed, prior to downstream analyses [11] or in which the refractive error in only the right eye was considered. The table below lists the number of variants identified in each step of the analysis for these analyses, which are labelled #1-4. The Venn diagrams display the degree of overlap for the variants identified in Step 1 (panel A) and in Step 2 (panel B).

The original analysis presented in the main text (analysis #2) and all 3 sensitivity analyses resulted in the identification of the same 6 SNP × *UniEdu* interactions in the Stage-II sample.

**Analysis**	**Phenotype in Steps 1 and 2**	**Adjust for *UniEdu* in Step 1** (GWAS)	**Adjust for *UniEdu* in Step 2** (Levene's test)	**Step 1 results:** Number of independent loci P < 1e-04	**Step 2 results:** Number of vQTL loci (Bonferroni-corrected)	**Step 3 results:** Number of SNP × *UniEdu* interactions in Stage-II sample (Bonferroni-corrected)
#1	*avMSE* in 2 eyes	-	-	956	29	6
#2	*avMSE* in 2 eyes	-	Yes	956	25	6
#3	*avMSE* in 2 eyes	Yes	Yes	911	23	6
#4	*MSE* in Right eye	-	Yes	910	28	6

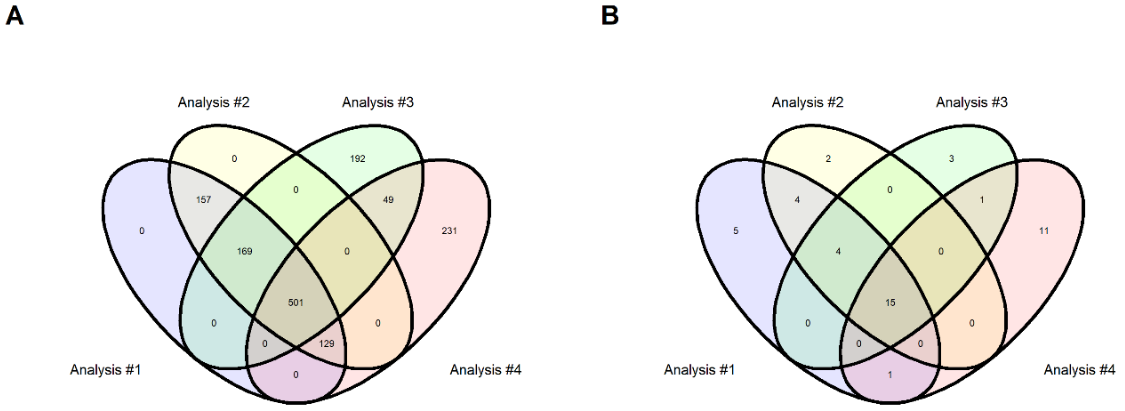

Box E. Supplementary Methods

Participants, phenotype and environmental variables

UK Biobank is a large prospective study examining the health and wellbeing of adults living in the United Kingdom (UK). The study had ethical approval from the National Health Service (NHS) Research Ethics Committee (Reference: 11/NW/0382). Signed and informed consent was obtained from all of the participants. Approximately 500,000 participants aged between 37 and 73 years attended a baseline assessment visit between 2006 – 2010 [1]. Data regarding educational attainment was collected using a structured interview during this baseline assessment. Participants were asked, "Which of the following qualifications do you have? (You can select more than one)". Those who did not report having a University or college degree were also asked "At what age did you complete your continuous full-time education?" We derived two variables based on these responses. The binary variable *UniEdu* was used to indicate whether or not individuals had a University or college degree and the integer variable *EduYears* was used to classify the age at which the participants completed their full-time education [2]. Participants who reported a school-leaving age of less than 15 years were assigned an *EduYears* of 15 years, while those who reported leaving school after the age of 21 years and those who held a University or College degree were assigned an *EduYears* of 21 years [2]. An ophthalmic assessment was included in the UK Biobank baseline visit only towards the later stages of recruitment. Approximately 23% of the participants underwent the ophthalmic assessment [3]. The refractive error phenotype (*avMSE*) was calculated as the average value across both eyes of the spherical equivalent (sphere + 0.5 × cylinder) refractive error from repeat autorefraction readings (Tomey RC 5000 instrument; Tomey GmbH Europe, Erlangen-Tennenlohe, Germany). All participants were asked their age-of-onset of spectacle (or contact lens) wear. We used this self-reported age-of-onset of spectacle wear as a continuous variable, *AOSW*, to indirectly quantify participants' refractive error, because of its known correlation with refractive error [4]. Blood samples were collected as part of the UK Biobank project. DNA was extracted and genotyped using either the UK BiLEVE Axiom array or the UK Biobank Axiom Array, as described [5]. Imputation was carried out with the IMPUTE4 program (https://jmarchini.org/software/), with a combined Haplotype Reference Consortium (HRC) reference panel and a merged UK10K/1000 Genomes phase 3 reference panel, as described [5].

A 'Stage-I' sample of unrelated participants of European ancestry with a valid *avMSE* information was selected (there were too few participants of non-European ancestry to study GxE interactions in other ancestry groups). Participants were excluded if they reported any of the following: cataracts, history of cataract surgery, corneal graft surgery, laser eye surgery, any other eye surgery in the last 4 weeks, "serious eye problems" or "eye trauma". In addition, individuals were excluded if their hospital records indicated a history of cataract surgery, eye surgery, retinal surgery or retinal detachment surgery. Participants were also excluded if they were from an assessment center that recruited 50 or fewer participants. Participants were classified as myopic if they had an *avMSE* ≤ -0.50 D [6]. From amongst these participants, the maximal set of unrelated participants was chosen using the R package *igraph* [5], which resulted in a final sample size for the Stage-I sample of N = 88,334. A 'Stage-II' sample was selected comprising of European-ancestry participants who were unrelated to each other, unrelated to any person in the Stage-I sample, and who had information available for *AOSW*, *UniEdu* and *EduYears*. This provided a Stage-II sample of N = 252,838. Participants in the Stage-II sample were classified as myopic if they had an *AOSW* greater than 5 years and less than or equal to 25 years [7].

Two-step screening strategy for identifying putative GxE interaction variants

The first screening step (Figure 1A) was a standard GWAS for the phenotype *avMSE* in the Stage-I sample of N = 88,334 participants, using a linear regression analysis implement with BOLT-LMM [8]. Sex, age, age-squared, a binary indicator of the genotype array (UK BiLEVE Axiom or UK Biobank Axiom array) and the first 10 ancestry principal components (PCs) were included as covariates. For this and all of the other analyses undertaken, non-binary covariates were standardized to have a mean of zero and a standard deviation of one, in order to facilitate model fitting. Imputed

genetic variants were included if they had a missing rate < 5%, a minor allele frequency (MAF) > 5% and a Hardy-Weinberg equilibrium test $P > 1 \times 10^{-6}$, which yielded approximately 7 million variants in total. Individuals with a missing genotype rate ≥ 2% were excluded. Independently associated SNPs in each region were selected by p-value-based clumping with PLINK [9] with a physical distance threshold of 500kb and a linkage disequilibrium (LD) r^2 threshold of 0.01. We applied a lenient p-value threshold ($P < 1 \times 10^{-4}$) for association with *avMSE* to identify SNPs to take forward to the second screening step. This p-value threshold was arbitrarily chosen in a pre-specified analysis plan, to provide a balance between specificity and sensitivity.

The second screening step (Figure 1B) was a variance heterogeneity analysis for the phenotype *avMSE* in the Stage-I sample (N = 88,334), using Levene's median test implemented with the software package OSCA [10]. The 956 genetic variants independently associated with *avMSE* that were identified in step 1 were taken forward for testing in step 2. We first carried out a linear regression analysis in R for the outcome variable *avMSE*, with *UniEdu*, sex, age, age-squared, genotype array and the first 10 PCs as predictor variables. As reported by Zhang et al. [11], inclusion of *UniEdu* in this step avoids inflation of false-positives when later testing for SNP × *UniEdu* interactions. The *avMSE* residuals from this model were then used as the phenotype for Levene's median test. A Bonferroni correction for multiple comparisons was applied to the alpha value for this test ($\alpha = 0.05/956 = 5.23 \times 10^{-5}$). Applying Levene's test required the use of "hard-called" genotypes. Bycroft et al. [5] reported that SNPs with MAF >5% in the UK Biobank study were imputed very accurately (imputation quality "INFO" metric >0.98). This meant that genotype uncertainty was unlikely to have adversely affected the results of Levene's test.

Gene-environment interaction tests

Gene-environment interaction tests were performed in R [12]. To test if any of the N = 25 SNPs identified using the 2-step screening strategy had evidence of an interaction with educational attainment, we carried out a formal test for genotype × education interaction in the independent sample of participants from the Stage-II sample (N = 252,838). Specifically, we fit a linear regression model with an interaction term for each variant in turn, as follows:

$$AOSW = \delta_0 + \delta_1 SNP + \delta_2 UniEdu + \delta_3 SNP \times UniEdu + \gamma C + \pi \qquad (Eq.\ \text{S1})$$

Where, *AOSW* is a $n \times 1$ vector of age-of-onset of spectacle wear values in the *n* participants in the Stage-II sample, *SNP* is a $n \times 1$vector of SNP genotypes (counts of the minor allele, coded 0, 1 or 2), *UniEdu* is a $n \times 1$ vector binary (0,1) variable indicating the absence or presence of University degree, *C* is a $n \times k$ matrix of covariates (age, age-squared, genotyping array, and the first 10 ancestry PCs; with non-binary covariates standardized to have a mean of zero and a standard deviation of one), γ is a $1 \times k$ vector of regression coefficients, and π is a residual. δ_0 is an intercept, while δ_1, δ_2 and δ_3 are the regression coefficients for the marginal effect for the SNP, the marginal effect for *UniEdu* and the SNP × *UniEdu* interaction effect, respectively. A Bonferroni correction for multiple comparisons was applied to identify δ_3 terms showing evidence of association, using an alpha value for this test of $\alpha = 0.05/25 = 0.002$.

Analogous linear regression models were fitted to test for genotype-by-*EduYears* interaction. However, as *EduYears* is a continuous exposure and its main effect on the outcome could be nonlinear, p-values and standard errors for all tests involving SNP × *EduYears* interactions were calculated using a robust Huber-White sandwich estimator (R package *estimatr*, available from https://github.com/DeclareDesign/estimatr):

$$AOSW = \delta_0 + \delta_1 SNP + \delta_2 EduYears + \delta_3 SNP \times EduYears + \gamma C + \nu \qquad (Eq.\ \text{S2})$$

Logistic regression models of the same form were applied to test for genotype-by-interaction effects associated with myopia status, for the outcome variable *Myopic* (1 = myopic, 0 = non-myopic). Linear

regression tests for a SNP × *UniEdu* interaction and SNP × *EduYears* interaction of the same form were carried out for the *avMSE*, *AOSW* and *Myopic* phenotypes in the Stage-I sample.

Gene-gene interaction tests

SNP × SNP interaction tests were performed to examine if any pair of SNPs from amongst the 25 SNPs identified using the 2-step screening strategy had evidence of a genotype × genotype interaction. A linear regression model with an interaction term was fitted for each pair of variants in turn, as follows:

$$AOSW = \delta_0 + \delta_1 SNP1 + \delta_2 SNP2 + \delta_3 SNP1 \times SNP2 + \gamma C + \pi \qquad (Eq.\ \mathrm{S3})$$

$$avMSE = \beta_0 + \beta_1 SNP1 + \beta_2 SNP2 + \beta_3 SNP1 \times SNP2 + \gamma C + \varepsilon \qquad (Eq.\ \mathrm{S4})$$

Where terms are defined as above. The *avMSE* phenotype was tested in the Stage-I sample and the *AOSW* phenotype was tested in the Stage-II sample. A Bonferroni correction for multiple comparisons was applied to identify δ_3 or β_3 terms showing evidence of association, using an alpha value for this test of $\alpha = 0.05/300 = 0.00017$ (accounting for a total of 25×25 / 2 tests).

Assessment of type-1 error rate and tests for gene-environment correlation

We carried out simulations to assess the type-1 error rate of Levene's median test when testing for variance heterogeneity with the *avMSE* phenotype in the Stage-I sample, and the type-1 error rate of linear regression when testing for a SNP × *UniEdu* interaction with the *AOSW* phenotype in the Stage-II sample. To test for gene-environment correlation, the following logistic regression model was fitted for each SNP:

$$\mathrm{logit}\, P(UniEdu = 1 \mid SNP, C) = \omega_0 + \omega_1 SNP + \gamma C + \varepsilon \qquad (Eq.\ \mathrm{S5})$$

As above, C is a $n \times k$ matrix of covariates (age, age-squared, genotyping array, and the first 10 ancestry PCs), γ is a $1 \times k$ vector of regression coefficients, and ε is a residual. ω_0 is an intercept. The ω_1 term quantifies the association between the SNP genotype and having a University degree.

Fig A. Manhattan plot of the results from the GWAS for refractive error (*avMSE*) in the Stage-I sample. The red horizontal line indicates the arbitrarily chosen p-value threshold ($P < 1 \times 10^{-4}$) used to select SNPs to take forward to the next stage of the analysis.

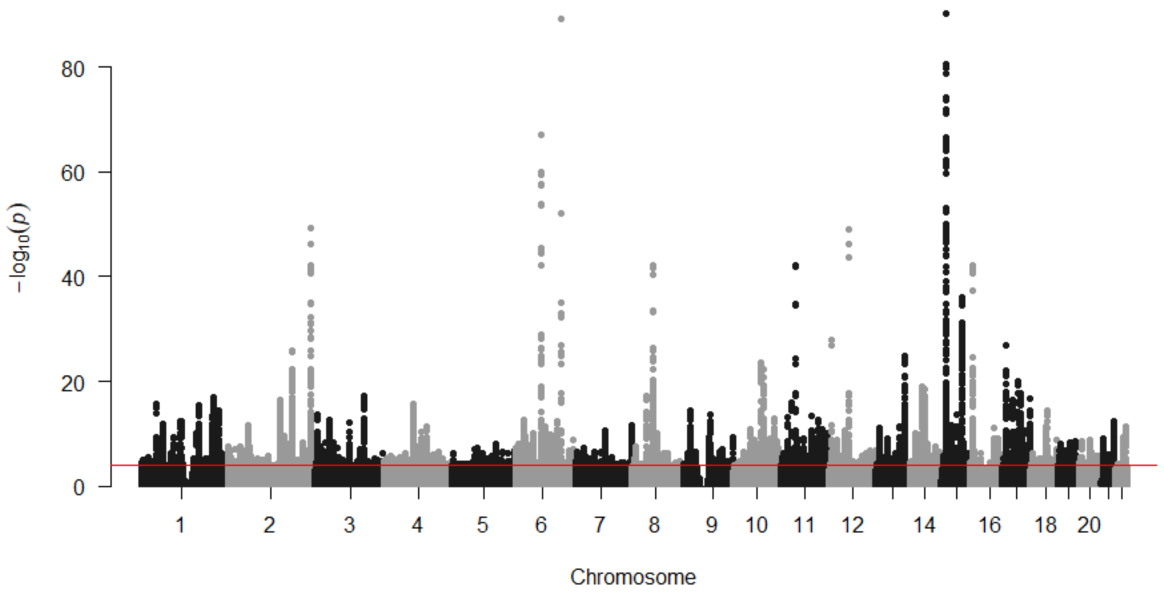

Fig B. SNP genotype-by-University education (GxE) interactions associated with age-of-onset of spectacle wear (*AOSW*). University education (*UniEdu*) was coded as a binary exposure. The nearest gene to the SNP is indicated above the SNP rsID. Error bars are 95% confidence intervals. SNPs with significant GxE interaction effects (P < 0.05/25) are shown in red/blue. The SNP risk allele was defined as the myopia-predisposing allele in a marginal SNP effects analysis.

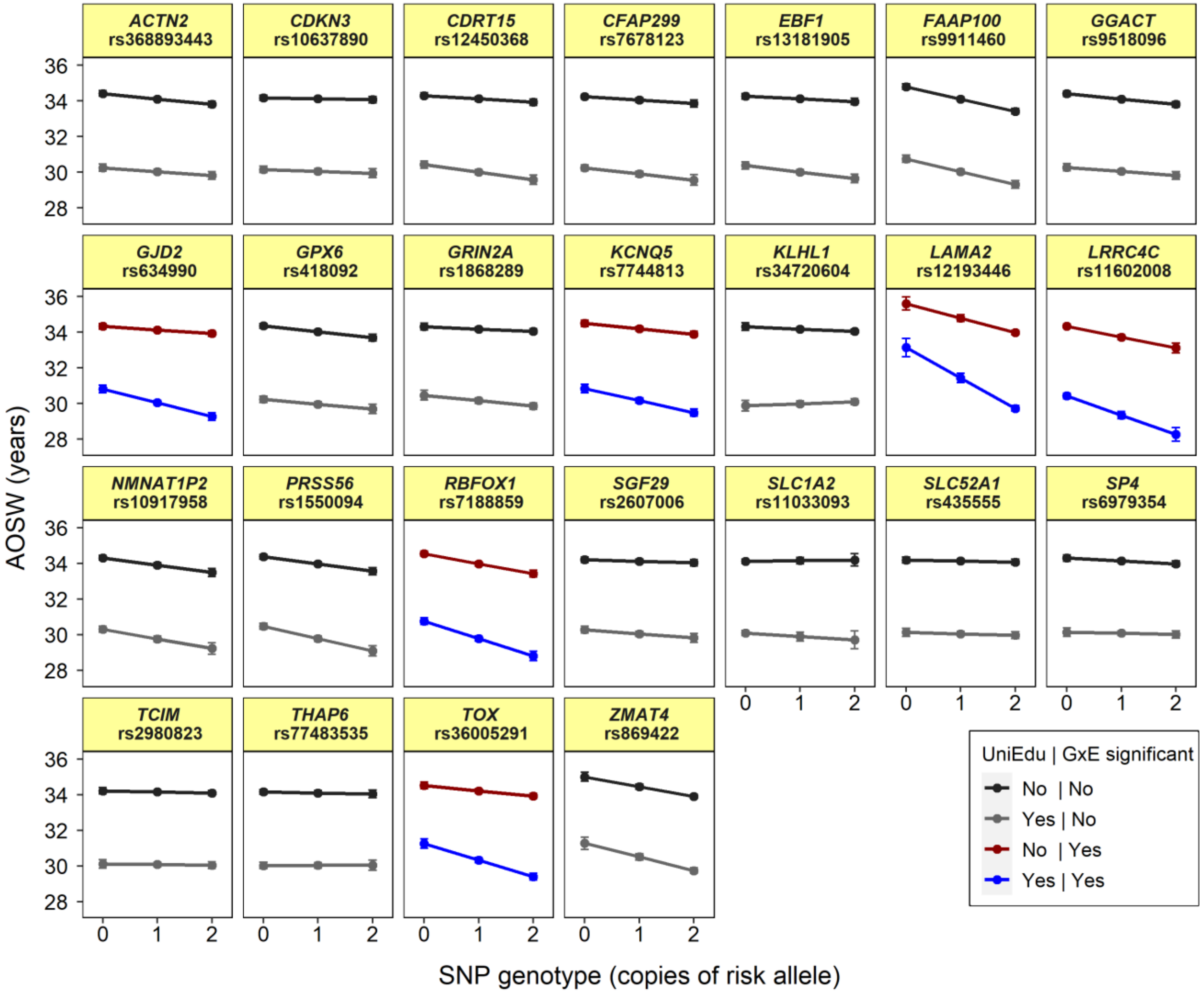

Fig C. Summary of the evidence for SNP × SNP interactions contributing to myopia development. Each tile indicates the statistical evidence (p-value) for a test of GxG interaction. Tests were carried out for the phenotype *avMSE* in the Stage-I sample and the phenotype *AOSW* in the Stage-II sample. A p-value of P < 0.00017 corresponds to a correction for 300 tests (α = 0.05/300, where 25×25 / 2 = 300).

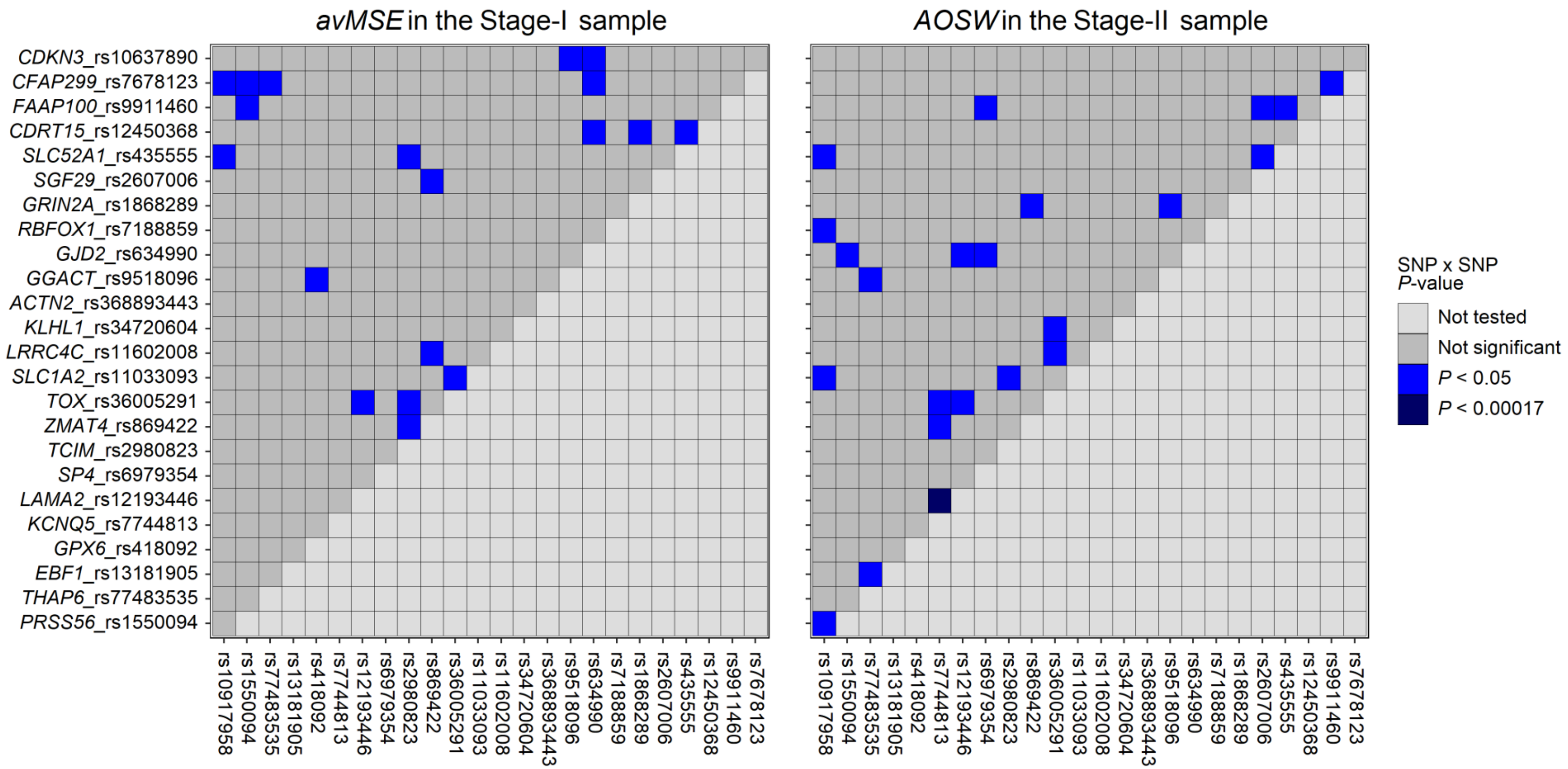

Supplementary References

1. Sudlow C, Gallacher J, Allen N, Beral V, Burton P, Danesh J, et al. UK Biobank: An Open Access Resource for Identifying the Causes of a Wide Range of Complex Diseases of Middle and Old Age. PLos Med. 2015;12(3):e1001779. doi: 10.1371/journal.pmed.1001779.

2. Mountjoy E, Davies NM, Plotnikov D, Davey Smith G, Rodriguez S, Williams CE, et al. Education and myopia: assessing the direction of causality by mendelian randomisation. BMJ. 2018;361:k2022. doi: 10.1136/bmj.k2022.

3. Cumberland PM, Bao Y, Hysi PG, Foster PJ, Hammond CJ, Rahi JS, et al. Frequency and Distribution of Refractive Error in Adult Life: Methodology and Findings of the UK Biobank Study. PLoS ONE. 2015;10(10):e0139780. doi: 10.1371/journal.pone.0139780.

4. Ghorbani Mojarrad N, Plotnikov D, Williams C, Guggenheim JA, U.K. Biobank Eye & Vision Consortium. Association Between Polygenic Risk Score and Risk of Myopia. JAMA Ophthalmol. 2020;138:7-13. doi: 10.1001/jamaophthalmol.2019.4421.

5. Bycroft C, Freeman C, Petkova D, Band G, Elliott LT, Sharp K, et al. The UK Biobank resource with deep phenotyping and genomic data. Nature. 2018;562(7726):203-9. doi: 10.1038/s41586-018-0579-z.

6. Flitcroft DI, He M, Jonas JB, Jong M, Naidoo K, Ohno-Matsui K, et al. IMI - Defining and Classifying Myopia: A Proposed Set of Standards for Clinical and Epidemiologic Studies. Invest Ophthalmol Vis Sci. 2019;60(3):M20-M30. doi: 10.1167/iovs.18-25957.

7. Guggenheim JA, Clark R, Cui J, Terry L, Patasova K, Haarman AEG, et al. Whole exome sequence analysis in 51,624 participants identifies novel genes and variants associated with refractive error and myopia Hum Mol Genet. 2022;31(11):1909-19. doi: 10.1093/hmg/ddac004.

8. Loh P-R, Kichaev G, Gazal S, Schoech AP, Price AL. Mixed-model association for biobank-scale datasets. Nat Genet. 2018;50(7):906-8. doi: 10.1038/s41588-018-0144-6.

9. Chang CC, Chow CC, Tellier LC, Vattikuti S, Purcell SM, Lee JJ. Second-generation PLINK: rising to the challenge of larger and richer datasets. GigaScience. 2015;4:7. doi: 10.1186/s13742-015-0047-8.

10. Wang H, Zhang F, Zeng J, Wu Y, Kemper Kathryn E, Xue A, et al. Genotype-by-environment interactions inferred from genetic effects on phenotypic variability in the UK Biobank. Sci Adv. 2019;5(8):eaaw3538. doi: 10.1126/sciadv.aaw3538.

11. Zhang P, Lewinger JP, Conti D, Morrison JL, Gauderman WJ. Detecting Gene-Environment Interactions for a Quantitative Trait in a Genome-Wide Association Study. Genet Epidemiol. 2016;40(5):394-403. doi: 10.1002/gepi.21977.

12. R Development Core Team. R: A language and environment for statistical computing. Vienna, Austria: R Foundation for Statistical Computing; 2008.

13. Howe LJ, Tudball M, Davey Smith G, Davies NM. Interpreting Mendelian-randomization estimates of the effects of categorical exposures such as disease status and educational attainment. Int J Epidemiol. 2021:doi: 10.1093/ije/dyab208. doi: 10.1093/ije/dyab208.

14. Fan Q, Wojciechowski R, Ikram MK, Cheng CY, Chen P, Zhou X, et al. Education influences the association between genetic variants and refractive error: A meta-analysis of five Singapore studies. Hum Mol Genet. 2014;23(2):546-54. doi: 10.1093/hmg/ddt431.

15. Wojciechowski R, Yee SS, Simpson CL, Bailey-Wilson JE, Stambolian D. Matrix metalloproteinases and educational attainment in refractive error: Evidence of gene–environment interactions in the Age-Related Eye Disease Study. Ophthalmology. 2012;120(2):298-305. doi: 10.1016/j.ophtha.2012.07.078.

16. Kiefer AK, Tung JY, Do CB, Hinds DA, Mountain JL, Francke U, et al. Genome-wide analysis points to roles for extracellular matrix remodeling, the visual cycle, and neuronal development in myopia. PLoS Genet. 2013;9(2):e1003299. doi: 10.1371/journal.pgen.1003299.

17. Hysi PG, Wojciechowski R, Rahi JS, Hammond CJ. Genome-wide association studies of refractive error and myopia, lessons learned, and implications for the future. Invest Ophthalmol Vis Sci. 2014;55(5):3344-51. doi: 10.1167/iovs.14-14149.

18. Patasova K, Khawaja AP, Wojciechowski R, Mahroo OA, Falchi M, Rahi JS, et al. A genome-wide analysis of 340 318 participants identifies four novel loci associated with the age of first spectacle wear. Hum Mol Genet. 2022. doi: 10.1093/hmg/ddac048.

19. Asefa NG, Neustaeter A, Vehof J, Nolte IM, Snieder H, Jansonius NM. Development and validation of a questionnaire-based myopia proxy in adults: the LifeLines Cohort Study. Br J Ophthalmol. 2022. doi: 10.1136/bjophthalmol2021-319166.

20. Pozarickij A, Williams C, Guggenheim JA, U.K. Biobank Eye and Vision Consortium. Non-additive (dominance) effects of genetic variants associated with refractive error and myopia. Mol Genet Genom. 2020;295(4):843-53. doi: 10.1007/s00438-020-01666-w.
